# Spatial Working Memory Deficits in Male Rats Following Neonatal Hypoxic Ischemic Brain Injury Can Be Attenuated by Task Modifications

**DOI:** 10.3390/brainsci4020240

**Published:** 2014-04-02

**Authors:** Amanda L. Smith, Courtney A. Hill, Michelle Alexander, Caitlin E. Szalkowski, James J. Chrobak, Ted S. Rosenkrantz, R. Holly Fitch

**Affiliations:** 1 Behavioral Neuroscience Division, Department of Psychology, University of Connecticut, 406 Babbidge Road, Unit 1020, Storrs, CT 06269, USA; E-Mails: CBodge@wihri.org (C.A.H.); caitlin.szalkowski@gmail.com (C.E.S.); james.chrobak@uconn.edu (J.J.C.); roslyn.h.fitch@uconn.edu (R.H.F.); 2 Division of Neonatology, Department of Pediatrics, University of Minnesota, 516 Delaware Street S.E. Minneapolis, MN 55454, USA; E-Mail: alex0213@umn.edu; 3 Department of Pediatrics, University of Connecticut Health Center, 263 Farmington Avenue, Farmington, CT 06030, USA; E-Mail: Rosenkrant@nso1.uchc.edu

**Keywords:** hypoxia ischemia, working memory, rodent model, eight-arm radial water maze, impulsivity

## Abstract

Hypoxia-ischemia (HI; reduction in blood/oxygen supply) is common in infants with serious birth complications, such as prolonged labor and cord prolapse, as well as in infants born prematurely (<37 weeks gestational age; GA). Most often, HI can lead to brain injury in the form of cortical and subcortical damage, as well as later cognitive/behavioral deficits. A common domain of impairment is working memory, which can be associated with heightened incidence of developmental disorders. To further characterize these clinical issues, the current investigation describes data from a rodent model of HI induced on postnatal (P)7, an age comparable to a term (GA 36–38) human. Specifically, we sought to assess *working* memory using an eight-arm radial water maze paradigm. Study 1 used a modified version of the paradigm, which requires a step-wise change in spatial memory via progressively more difficult tasks, as well as multiple daily trials for extra learning opportunity. Results were surprising and revealed a small HI deficit *only* for the final and most difficult condition, when a delay before test trial was introduced. Study 2 again used the modified radial arm maze, but presented the most difficult condition from the start, and only one daily test trial. Here, results were expected and revealed a robust and consistent HI deficit across all weeks. Combined results indicate that male HI rats can learn a difficult spatial working memory task if it is presented in a graded multi-trial format, but performance is poor and does not appear to remediate if the task is presented with high initial memory demand. Male HI rats in both studies displayed impulsive characteristics throughout testing evidenced as reduced choice latencies despite more errors. This aspect of behavioral results is consistent with impulsiveness as a core symptom of ADHD—a diagnosis common in children with HI insult. Overall findings suggest that task specific behavioral modifications are crucial to accommodating memory deficits in children suffering from cognitive impairments following neonatal HI.

## 1. Introduction

Infants who undergo birth complications, such as prolonged labor or cord prolapse, are at a high risk for hypoxic ischemic (HI) injury [1,2,3,4]. Such injuries in the term infant typically lead to a diffuse form of brain injury, termed hypoxic ischemic encephalopathy (HIE; [1,5,6]). HIE reflects the evolution of a cascade of molecular events triggered by the insult, leading to immediate as well as delayed cell death and neural tissue loss, with gross pathology emerging over a period of 3–4 days [1,7]. Additionally, another risk factor for neonatal HI is prematurity (<37 weeks gestational age (GA)) and/or very low birthweight (VLBW; <1500 g [1,8,9]). In contrast to the pathology in term infants, HI events in premature infants lead to cell death primarily in the germinal matrix containing glial precursor cells [1,10,11,12,13]. In this population, injury typically manifests as white matter damage due to periventricular leukomalacia (PVL), which is a form of non-hemorrhagic injury [8,14,15,16]. Intracranial hemorrhage can also occur in this population as evidenced by intraventricular (IVH) and/or periventricular (PVH) hemorrhages [6,10]. Overall, in both term and preterm infants, the severity and timing of an HI insult determines the extent of resulting neural damage [17].

Though the incidence rate of HI insult is much lower in term as compared to preterm infants (approximately 0.2%–0.4% of term *vs*. 60% of VLBW/preterm births; [1,2,6,18,19,20]), both populations undergo similar events leading to cell death and tissue damage in the brain [21,22,23]. The cascade of deleterious events that eventually lead to cell death is characterized in the term infant by diffuse gray matter damage (including hippocampal, basal ganglia, and cortical volume reduction; [1,6,18,21,22,24,25]), although white matter motor tracts can also be affected [26]. This contrasts the more focal and predominantly white matter injuries seen in preterms with HI.

Due to medical advances, more infants with HI now survive than in the past, leading to an increase in the numbers of children with associated behavioral deficits later in life [2,27,28,29,30,31]. Indeed, despite the varying etiologies noted above, both term and preterm HI populations show increased frequencies of language deficits [32,33,34,35,36,37], memory impairments [38,39,40,41] and developmental disorders such as ADHD [31,42,43,44,45,46,47].

Of particular importance are the memory impairments seen in term and preterm children who suffer an HI insult, in part, because they are often related to additional impairments such as inattention, which is a common symptom of ADHD [42,48,49,50]. It has been suggested that cognitive and academic difficulties reflecting memory impairments are more common than motor, visual, or hearing impairments in preterm children that undergo an HI insult [51,52]. In one study exploring spatial location memory in extremely low birth weight (ELBW; <1000 g) children, extreme prematurity (and the subsequent injuries that follow) were shown to be a risk factor for memory deficits [40]. Similarly, Espy *et al*. (2002) [53] showed specific working memory deficits in a premature population, but this time on a task incorporating a delay. Lower IQ scores related to memory impairments have also been reported in VLBW populations (e.g., deficits on digit span tasks; [54,55,56,57]). While literature on behavioral outcomes following an HI insult specifically in term infants is limited, it has been reported that severely HI injured term infants display deficits on “everyday memory tasks” compared to infants suffering from a mild HI birth insult [33].

In addition to memory impairments following an HI insult, deficits in attention or instances of hyperactive behavior are often seen concurrently [50], and diagnoses of ADHD, autism and other developmental disorders are often associated with memory and executive functioning impairments [31]. A common characteristic in children who suffer an HI insult and are diagnosed with ADHD is impulsive behavior. Though data on term infants who display memory impairments and impulsive characteristics is scant, there are many studies assessing neurodevelopmental impairments in preterm children. Since both populations undergo similar forms of brain damage following HI, it is therefore not surprising that these populations exhibit highly overlapping developmental outcomes. For instance, preterm children who were tested on a working memory task displayed increased rates of inattention, and overactive/impulsive behavior that occurred along with working memory impairments [50]. Also, preterm children (particularly males) scored higher than age-matched controls on hyperactivity scores, and showed higher rates of externalizing hyperactive behavior [58,59,60,61,62]. Children born at almost term who suffered birth complications also showed heightened risk of ADHD and/or hyperactive tendencies, as reported by teachers and parents [63]. Problems are also seen in the HI population for executive functioning, which includes poor planning and problem solving [54,55,56,64,65,66]. For example, after investigating a national cohort of children born prematurely, parents and teachers of these children reported problems in all executive functioning areas that were assessed (*i.e.*, attention, hyperactivity, planning/organizing, and working memory; [67]).

It has been postulated that impairments in memory domains in HI populations might reflect damage to the hippocampus, the striatum and associated projection targets, and/or the dorsolateral prefrontal cortex [38,53,68,69]. In regards to the brain area that could relate to attention deficits, it has been suggested that caudate damage could be involved [62]. All of these neuropathologies are frequently observed to varying degrees in neonatal HI populations.

To address these important clinical issues, animal research has been used to assess behavioral deficits associated with induced neonatal HI injury. The experimental assessment of long term deficits following an HI injury can facilitate both our understanding of the mechanisms underlying outcomes, as well as providing a platform to assess potential interventions and treatments for efficacy. Interestingly, while the majority of clinical literature focuses on preterm infants who undergo an HI insult, most of the animal literature uses a P7 model of HI injury—which roughly equates to term HI injury (*i.e.*, HIE; [70,71]). Specifically, when this injury is induced in the P7 rat (via artery cauterization/exposure to reduced oxygen), it produces a pattern of neuropathology that includes decreased volumes of cortex, hippocampus, and corpus callosum, as well as ventriculomegaly [72,73]. Behavioral impairments (such as memory deficits) associated with P7 HI damage have also been reported. Our lab, for example, has previously shown deficits on a Morris water maze task of spatial learning and memory [74,75,76,77,78] an effect also reported by others [21,79,80,81,82,83,84,85]. Researchers using the P7 HI rat model have also shown learning and memory deficits on a plus maze, as well as a standard eight-arm radial land maze task [86,87,88,89]. On studies using the radial arm land maze, which requires intact working and reference memory, the impairment in HI rats seems to be most pronounced early in testing [86], but other researchers have shown that deficits are progressive over the lifespan (*i.e.*, memory performance gets worse over time after the initial HI insult, with P7 HI rats showing a more severe impairment on the radial arm land maze at 16 weeks compared to seven weeks post insult; [89]). This P7 HI model has also revealed attentional impairments (in the form of impulsive characteristics), most often seen on a choice reaction time (CRT) task and also on operant fixed interval extinction tasks [86,90], further relating an HI injury to ADHD-like behavior.

Based on the above clinical and animal literature revealing the debilitating effects of neonatal HI injury, the current study sought to further explore working memory impairments in a P7 rodent model of HI injury—specifically relating to task design and demands. Note that in the animal literature, working memory is defined as “a short term memory for an object, stimulus, or location that is used within a testing session, but not typically between sessions” [91]. A classic task used to assess working memory in rodents is the radial arm land maze (see seminal work by Olton and Honig; [92,93,94]), and as noted above, this task has previously been used to show working memory deficits in P7 HI rats (e.g., [86,89]). Here, we wanted to establish a baseline characterization of working memory impairments in P7 HI rats using a novel modified (match-to-sample) radial arm water maze [95]. This task differs from and is more difficult than a standard radial arm water maze, and difficulty level can be increased by addition of a delay component between sample and test trials. In Study 1, the task was initially presented at an easy level (three arms open), and became more difficult as testing progressed, with the last two weeks of testing utilizing all eight arms of the radial arm water maze, as well as a delay. Also, multiple daily trials were provided in Study 1. However, only trial 1 errors are reported since the first trial carries the most working memory demand (with subsequent trials providing reinforcement. We hypothesized that HI animals would display significant memory impairments as compared to sham animals on most weeks of testing. The results from this study did not support our hypothesis, and in fact showed that HI animals had the ability to perform comparably to shams when provided a step-wise multi-trial version of the task (at least until the delay was introduced, when a small HI deficit was seen). In Study 2, the more difficult version of the task where all arms were open and a delay interval was implemented from the start, and only one daily trial was provided. Again we hypothesized that male HI rats would be impaired on all weeks of testing as compared to sham rats, and results on the more difficult task version confirmed a persistent HI deficit across all weeks (even with an extra four weeks of testing relative to Study 1), with no evidence of remediation.

## 2. Experimental Section

### 2.1. Subjects (Studies 1 and 2)

In both studies, subjects consisted of male Sprague-Dawley rats born to time-mated dams (Charles River Laboratories, Wilmington, MA; Study 1 *n* = 24; Study 2 *n* = 24). Dams were shipped to the University of Connecticut on embryonic Day 5 (E5) to avoid prenatal shipping stress. Dams/litters were housed in the University of Connecticut Bousfield vivarium, on a 12-h light/dark cycle. Pups were born on E22, and culled to litters of 10 (eight males and two females) on P1. Only male subjects were used, based on prior evidence that behavioral deficits are more robust in male as compared to female HI rats [74,77,96]. Upon weaning (P21), subjects were pair-housed with like-treated littermates (to reduce stress of weaning), and received food and water *ad libitum*. Subjects were shifted to single housing for ease of behavioral testing in adulthood (P70). The University of Connecticut Animal Care and Use Committee approved all procedures.

### 2.2. Surgical Procedure (Studies 1 and 2)

On P7, pups were randomly assigned to receive sham or HI surgery. Pups were anesthetized with isoflurane (2.5%) and a vertical incision was made on the neck. Pups assigned to the HI group had the right common carotid artery located, separated from surrounding tissue, and cauterized to restrict blood flow. Sham animals also received a vertical incision on the neck, but were sutured immediately after. All animals received footpad injections for identification. To maintain normal body temperature, animals in Study 1 were placed under a warming lamp for recovering from anesthesia, while pups from Study 2 were placed in a temperature regulated incubator to recover from anesthesia (due to receipt of new equipment). After a 2-h recovery and feeding period, pups were returned to their dams to nurse. After feeding, Study 1 HI pups were placed in an airtight container under a heating lamp, and were exposed to 8% oxygen (balanced with nitrogen) for 120 min. Study 1 sham animals were placed in a similar container under a heating lamp, but were subjected to room air for 120 min. Study 2 HI and sham animals received a similar procedure, but both containers were also placed on top of a temperature-controlled slide warmer (along with an overhead heating lamp). Following hypoxia, all animals were returned to their dams until weaning on P21.

### 2.3. Behavioral Testing (Study 1)

**Figure 1:**
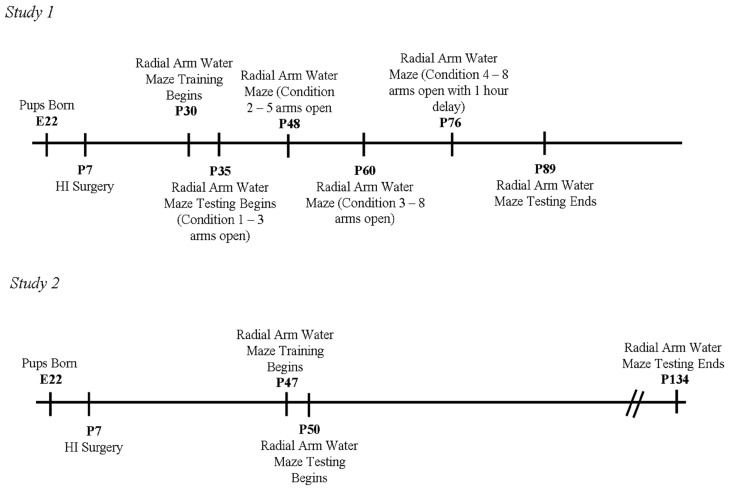
Study 1 and Study 2 Timelines.

Pre-testing began on P30, with two days of radial arm water maze training (refer to Figure 1 for a timeline of experimental testing procedures). A round 122-cm. diameter black Plexiglas pool was used, housing a black metal radial arm water maze. The radial arm water maze consisted of eight removable steel arms that could be blocked off or opened for testing. The pool was filled with room temperature water, with a removable black plastic platform submerged beneath the water. This acted as the escape platform (reinforcer), and could be positioned in any arm. The test room contained spatial cues, such as a small floor lamp in one corner, a table on the opposite side, two empty walls, and a cage rack opposite one of the empty walls.

Initial training was performed for two days, allowing the animals to get accustomed to the testing procedure, and ensuring they had no baseline impairments in traversing the maze. During the two days of training, four trials were provided. To begin, the animal was placed in the water at the end of a start arm. The submerged platform was placed at the end of another “goal” arm. All other arms, except for the start and goal arms, were blocked off. Subjects were required to swim out of the start arm, navigate to find the open goal arm, and mount the submerged platform. After the subject found the goal arm, it was taken out of the water, dried off with a towel, and returned to a “holding cage” on a table in the room. Since early testing was performed while animals were pair-housed, the cage-mate was given the same test with the same goal and start arm, after his mate had finished. The same goal and start arm were used for each of the four trials, and latency to the goal was recorded. This procedure was repeated on the following day, but different start and goal arms were used (for a detailed description of the task, see [95]).

#### 2.3.1. Weeks 1 and 2 (Condition 1—3 Arms Open)

At P35, working memory assessment began (see Figure 1). Testing occurred over four days of a five-day testing week. Initially, all animals received a “forced sample” trial using the same procedure described above. This forced the animal to the goal arm, providing a representation of the goal arm in working memory. Once both cage-mates performed a forced sample trial, the first subject was given a test trial in which another arm was opened (*i.e.*, three open arms total—start arm, goal arm, and an additional “choice” arm). The goal arm was *always* the same as the forced sample trial, although the start arm varied. The same start arm position was never used for the sample and test trial, to ensure subjects employed spatial and not angle-of-turn information. Three additional test trials were given, always with the goal arm remaining the same but with the choice and start arm varying. Sequences of goal, start, and choice arms varied each of the eight days of testing for Weeks 1 and 2. On all trials, latencies to goal, total number of errors, and latencies per choice were recorded (data reported only for Trial 1).

Throughout testing weeks, control trials were administered on the fifth day of the test week. During this trial, animals were placed in a start arm and were required to find the goal arm with all arms open. Subjects were not “shown” the location of the goal arm, so they were not expected to know where it was. This ensured that all animals were equally able to swim and locate the platform in the absence of any memory demand.

#### 2.3.2. Weeks 3 and 4 (Condition 2—5 Arms Open)

On P48, five arms were opened in the maze: a goal arm, start arm, and three choice arms (noting that with more choices, the task was more difficult). Again, a forced sample was given at the beginning of a testing day. The four subsequent test trials consisted of the same three choice arms and goal arm, but the start arm varied between trials. Testing was performed four days a week for two weeks, with a control test on Day 5. As above, latencies to goal, number of errors, and latencies per choice were recorded for each animal for each trial.

#### 2.3.3. Weeks 5 and 6 (Condition 3—8 Arms Open)

On P60, all eight arms of the maze were open: a goal arm, start arm, and six choice arms (thus harder than previous testing weeks). Again, four test trials were given following a forced sample, and control trials were performed on the 5th day of each week. Data was recorded as in previous trials.

#### 2.3.4. Weeks 7 and 8 (Condition 4—8 Arms Open with 1 h Delay)

On P76, rather than test trials being administered directly after the forced sample, test trials were administered after a 1-h delay. All eight arms were open during the four test trials, throughout the eight days (two weeks) of testing. Again, a control trial was administered on the 5th day, and data was recorded as in previous trials.

### 2.4. Behavioral Testing (Study 2)

Radial arm water maze training for Study 2 began at P47, in the same room as Study 1 (see Figure 1 for study timeline). A slightly later starting age was used because of concern about P35 rats performing the more difficult task (with the extra 12 days expected to benefit both groups equally). That is, we determined that a slight increase in age might be beneficial to the animals since older animals have been shown to perform better on a working memory task [97,98]. Finally, the same Plexiglass tub filled with room temperature water, housing the same eight arm maze insert and submerged platform, were used.

In brief, all animals were again given radial arm water maze training in order to acclimate to the task and confirm adequate swimming ability, navigation, and ability to mount the platform (see Study 1). Following training, animals were tested on the working memory assessment four days a week for 12 weeks (note that an additional four weeks of testing were implemented to see if HI subjects could ever reach sham performance). At the beginning of each test day, animals again received a single forced sample trial, where all arms of the maze were blocked except for the start and goal arm. After locating the platform, subjects were removed from the pool and placed in their holding cage for 10 min before being given the test trial. During a test trial, all eight arms of the maze were open, the animal was placed in a different start arm, and the goal arm remained the same. Unlike Study 1, only one test trial was administered after a forced sample—again making the task more difficult than Study 1. As in Study 1, different start and goal arms were used each day, and sequences of start and goal locations varied systematically among forty-eight possible combinations. Number of errors and latency to first arm choice were recorded for each animal on each day. As in Study 1, animals received regular “control” trials to provide ongoing measures of baseline (chance) performance, and to validate comparability across treatments on non-memory components of the task (swimming, vision).

Since this version of the task included a pre-test delay from the very beginning of testing, the paradigm was comparable to the last two weeks of testing in Study 1 (Weeks 7 and 8). Note that although an hour delay was used in Study 1 *versus* a 10 min delay in Study 2, it has been shown that these delay intervals produce the same number of errors in rats, and thus are of approximately equal difficulty [95]. Exogenous manipulations known to impair working memory have also been shown to interfere equally with performance on this task utilizing a 10 or 60 min delay [95]. Other studies have also reported delay-dependent working memory deficits on a radial arm land maze task using delays up to 640 min [99,100] further indicating the dependence on working memory in a delay-dependent radial water maze task.

### 2.5. Histology (Studies 1 and 2)

At the completion of testing, animals were weighed and anesthetized with an i.p. cocktail of ketamine (100 mg/kg) and xylazine (15 mg/kg). Animals were flushed with 0.9% saline solution followed by 10% buffered formalin phosphate. Brains were removed from the skull, weighed and placed in a 10% formalin solution. In Study 1, a vibratome was used to slice brains at 60 μm, and every 5th section was mounted on a chrom-alum subbed slide in preparation for staining. Each section was stained for cell bodies using a Nissl stain. In Study 2, brains were placed in a 30% sucrose solution for cryoprotection prior to being sliced at 60 um using a cryostat. Every 3rd section was mounted on a chrom-alum subbed slide and was stained using a Nissl stain. For both studies, sections were then analyzed for cortical, hippocampal, and ventricular volume using Stereo Investigator Microbright field software on an Axio 2 Zeiss Microscope (Carl Zeiss, Thornwood, NY, USA). The cortex and hippocampus measurements were analyzed from approximately Bregma −2.12 mm to Bregma 6.04 mm, or when the hippocampus became visible on the slides until the dorsal and ventral hippocampus merged together. Ventricular measurements were analyzed from approximately Bregma 1.20 mm to Bregma −2.3 mm. Both of these measures are approximations due to variability in the quality of sections. Additionally, to achieve stereological validity, adequate sections were always counted to yield a coefficient of error less than 0.05. Volumes were quantified using 100× magnification with Cavalieri’s Estimator software and a grid overlay, and measurements were always performed blind to Treatment group.

### 2.6. Statistical Analyses (Studies 1 and 2)

All statistical analyses were performed using SPSS software (IBM, Armonk, NY, USA) with an alpha criterion of 0.05, two-tailed, unless otherwise stated. Behavioral results from Study 1 (*n* = 24) and Study 2 (*n* = 24) were analyzed separately, with the exception of a *post hoc* comparison between mean weekly errors for Weeks 7 and 8 (when comparable tasks were used), across Study. For each Study, in addition to analyzing test trial errors over Weeks/Conditions, we analyzed latency/errors for control trials to ascertain Treatment effects on the task in the absence of memory demands.

#### 2.6.1. Study 1 and 2, Anatomical Analyses

In both studies, volumetric measures of the right and left hippocampus, cortex, and ventricles were separately analyzed by multi-variate ANOVA (Hemisphere (Within, two levels) and Treatment (Between, two levels)), and again using Hemisphere as a within variable (two levels, right *vs*. left) but within each Treatment group separately. Independent samples *t*-tests were performed (with Treatment as the between), on right cortical and right hippocampal volumes. For reasons explained below, anatomic data from Study 1 was further separated using a cortical atrophy measure (right/left ratio score) and, following this separation, volumetric values were compared across Studies via multi-variate ANOVAs and *t*-tests.

#### 2.6.2. Study 1, Error and Latency Analyses

Trial 1 errors (by day) from Study 1 were analyzed using multi-variate ANOVA with Treatment (between, two levels), Condition (within, four levels), Week (within, two levels) and Day (within, four levels). We found no Day interactions with Treatment or Condition, thus final (reported) analyses collapse over Day, and use mean Trial 1 errors for each Week as the dependent variable. A comparison of Week 1 *vs*. 2 scores within each Condition was used to assess learning (*t*-tests at each Condition corrected with Tukey’s tests, with Week as the between variable, and performed within each Treatment group separately). The comparison of second to first week scores within each Condition was based on a priori hypothesis that fewer errors should be seen in the second week if learning occurs. Also, *t*-tests corrected for multiple comparisons with Tukey’s test were run for each Week on mean errors, using Treatment as the between variable. (Since we have previously reported a memory deficit in HI rats on the Morris water maze, these particular *t*-tests were one-tailed; [74,75,101]). Finally, average latencies per choice for Trial 1 were calculated by taking total latency to goal, divided by the number of erroneous choices + 1 (goal arm). This measure yields an index of time to make each arm choice, since total time is divided by number of arms entered (error + correct). Short latencies to make correct choices (*i.e.*, on few or no error trials) could indicate robust memory and performance, while longer latencies are typically expected when errors are made. Conversely, short latencies on high error trials could reflect impulsivity (though additional factors can influence latency times). A multi-variate ANOVA was performed on weekly means for these scores (in seconds), using Treatment as a between variable (HI and sham; two levels), and Condition (four levels) and Week (two levels) as within variables. Individual *t*-tests, again performed with Tukey’s test to correct for multiple comparisons were also used to assess Treatment effects on latency for each Week.

#### 2.6.3. Study 2, Error and Latency Analyses

For Study 2, test conditions did not vary (only one condition was used). Thus errors per trial across days within the 12 testing weeks were averaged into six Blocks, with scores for each Block representing mean errors for that 2-week test period. One HI animal was dropped from the study due to possible seizure activity, and this subject was not included in any behavioral/anatomical analyses. A 6 (Block) × 2 (Treatment) repeated measures ANOVA was then performed on mean errors. Also, paired samples *t*-tests between Block 1 and 6 were performed within each Treatment, to assess overall learning within each Treatment group. Finally, average latency per choice for each test trial was again calculated as total latency to goal divided by errors + 1 (goal arm). A 6 (Block) × 2 (Treatment) two-tailed repeated measures ANOVA was performed on mean weekly latency (in seconds), followed by individual *t*-tests, further corrected with Tukey’s test, comparing latency per choice within each Treatment, for each Block of testing.

#### 2.6.4. Studies 1 and 2, Cross-Study Error Analysis—Weeks 7 and 8 Only

Given differences in task demands, it was deemed statistically invalid to perform an analysis of overall errors across the two studies. However, since subjects received essentially the same task during Weeks 7 and 8, we did compare this subset of data. Notably, a small but potentially confounding between-study difference (eight arms and an hour delay was used in Study 1, eight arms and a 10 min delay was used in Study 2), if significant, would bias results against our hypothesis (*i.e.*, it could be argued that a harder task was used where we predicted better performance). Also, although data from Study 2 was initially assessed using mean errors for 2-week Blocks (described above), in this analysis we used mean error data from Weeks 7 and 8 separately, to provide comparability to Study 1. Specifically, a multi-variate ANOVA was performed using Study as a between variable (two levels), Treatment as a between variable (two levels), Week as a within variable (two levels), and mean errors per week as the dependent variable.

## 3. Results and Discussion

### 3.1. Anatomic Results (Studies 1 and 2)

#### 3.1.1. Study 1 Anatomy

For Study 1, an overall ANOVA on cortical volume scores (mm^3^) was performed, using Treatment (between, HI *vs*. Sham) and Hemisphere (within, right *vs*. left). No significant effect of Treatment (*F*(1,22) = 0.551, *p* > 0.05), Hemisphere (*F*(1,22) = 0.395, *p* > 0.05), nor interaction of Hemisphere × Treatment (*F*(1,22) = 0.089, *p* > 0.05) were found (although scores were in the expected direction). Similarly, an ANOVA for hippocampal volume (mm^3^) using Treatment (HI and Sham) and Hemisphere (right *vs*. left), also revealed a lack of significant effect of Treatment (*F*(1,22) = 1.148, *p* > 0.05), Hemisphere (*F*(1,22) = 0.484, *p* > 0.05), and Hemisphere × Treatment interaction (*F*(1,22) = 0.067, *p* > 0.05) (again, scores in expected direction). For ventricular volume, two animals were dropped (1 HI and 1 Sham) because their tissue did not provide reliable anatomic boundary measures. A similar ANOVA on ventricular volume (mm^3^) in HI and sham animals again revealed a lack of significant effect of Treatment (*F*(1,21) = 0.084, *p* > 0.05), Hemisphere (*F*(1,20) = 0.007, *p* > 0.05), and Hemisphere × Treatment interaction (*F*(1,20) = 0.525, *p* > 0.05).

#### 3.1.2. Study 2 Anatomy

**Figure 2:**
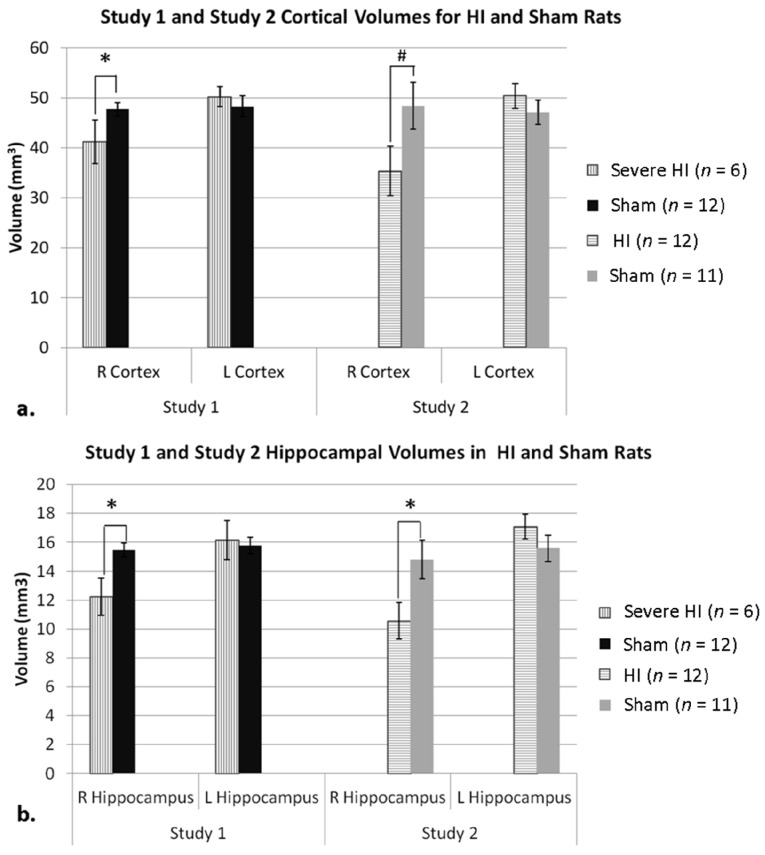
(**a**) As expected in Study 1 (since the Severe HI subset was selected for cortical atrophy), a *t*-test on right cortical volume revealed a significant effect of Treatment (* *p* < 0.05, Severe HI smaller than sham). For Study 2, an ANOVA comparing HI and sham animals revealed a Hemisphere × Treatment interaction, and a *t*-test also showed a trend (# *p* = 0.07) for HI to have smaller right cortical volume. Analysis of right cortical volume across Study confirmed a robust overall HI effect (*p* < 0.05) and a lack of between-study differences. (**b**) For Study 1, an ANOVA comparing hippocampal volumes in severe HI and sham animals revealed an overall Treatment effect (*p* = 0.05). A *t*-test comparing right hippocampal volume in both groups in Study 1 revealed a significant effect of Treatment (* *p* < 0.05), as was seen in Study 2 (* *p* < 0.05; HI smaller than sham). Analysis of right hippocampal volume across Study confirmed a robust overall HI effect (*p* < 0.005), and a lack of between-study differences.

For Study 2, a similar ANOVA on cortical volume did reveal a significant Hemisphere effect (*F*(1,21) = 5.776, *p* < 0.05), as well as a Hemisphere × Treatment interaction (*F*(1,21) = 8.159, *p* < 0.05), reflecting smaller right cortical volume in HI rats (Figure 2a). Therefore, an independent samples *t*-test on right cortical volume (using Treatment as the between) further revealed a trend for significance (*t*(21) = −1.923, *p* = 0.07; Figure 2a), with HI animals showing smaller right cortical volume compared to shams. A second repeated measures ANOVA on hippocampal volume also revealed a significant Hemisphere effect (*F*(1,21) = 11.016, *p* < 0.05), and a Hemisphere × Treatment interaction (*F*(1,21) = 6.756, *p* < 0.05). Again, an independent samples *t*-test on right hippocampal volume (using Treatment) revealed a significant effect (*t*(21) = −2.300, *p* < 0.05), with right hippocampus in HI animals significantly smaller than shams (Figure 2b). Finally, a repeated measures ANOVA on ventricular volume revealed a significant Hemisphere × Treatment interaction (*F*(1,21) = 8.557, *p* < 0.05), indicating different volumes in HI *versus* sham animals based on Hemisphere (data not shown).

#### 3.1.3. Study 1 Anatomy Re-Analysis—Sub-Grouping by Cortical Atrophy Measures

The failure to replicate significant anatomic effects in HI subjects from Study 1 (although scores were in the expected direction) was a matter of concern, since any comparison of behavioral data across studies assumes comparable damage. We considered the possibility that less HI damage occurred in Study 1 *versus* 2, due to a slight change in our surgical procedures (the acquisition of an incubator and slide warmers for Study 2)—particularly given evidence that cooling provides neuroprotection against HI injury [91,102,103,104,105,106]. Notably, Study 1 practices had been successfully employed in our lab for many years (with significant anatomic and behavioral HI effects reported; [86,87,88,107]). However, to directly test the possibility, we examined damage in HI subjects from Study 1 using a cortical atrophy measure. Specifically, the volume of the right cortex was divided by the volume of the left, to get a raw ratio in mm^3^. These atrophy (ratio) scores were then organized in ascending order and split into two groups (Mild and Severe, *n* = 6 each). Re-analysis of cortical volumes from Study 1 (mm^3^) using this revised Treatment designation (three levels: Severe HI (*n* = 6), Mild HI (*n* = 6), and sham (*n* = 12)) and Hemisphere (right *versus* left), now revealed a significant overall Hemisphere × Treatment interaction (*F*(2,21) = 5.667, *p* < 0.05; see Figure 2a), with a one-tailed independent samples *t*-test on right cortical volume in Severe HI *versus* sham animals showing a significant effect of Treatment (*t*(16) = 1.839, *p* < 0.05; Figure 2a). Noting that this effect might be expected since we had specifically chosen the subset of HI subjects with the most cortical damage, we also used the revised Treatment designation to re-examine ventricular and hippocampal volumes. Though no significant Treatment effects were seen for ventricular values, a repeated measures ANOVA revealed a subtle overall Treatment effect (*F*(1,16) = 4.363, *p* = 0.05) when comparing severe HI animals and shams in regards to hippocampal volume. Additionally, a one-tailed independent samples *t*-test revealed that severe HI animals displayed significantly smaller right hippocampal volumes compared to shams (*t*(16) = 1.990, *p* < 0.05; Figure 2b). Moreover, a new cross-Study repeated-measures ANOVA on both right cortical and hippocampal volume, but using Severe HI’s (Study 1) and all HI’s (Study 2) [Treatment (two levels; HI *vs*. sham), Hemisphere (two levels, right *vs*. left) and Study (two levels)] confirmed Treatment × Hemisphere interactions for both measures [(*F*(1,37) = 10.580, *p* < 0.005); (*F*(1,37) = 10.278, *p* < 0.005), respectively], although no main effects nor interactions with Study were seen (validating an assessment of the severe HI sub-group from Study 1 as comparable to HI rats in Study 2; Figure 2). On account of this finding, further independent samples *t*-tests were done to assess right cortical and hippocampal volume in HI *versus* sham animals using HI and sham animals from both studies combined. These results revealed significantly smaller right cortical and hippocampal volume in HI animals as compared to shams [(*t*(39) = 2.639, *p* < 0.05); (*t*(39) = 3.249, *p* < 0.005), respectively].

Moreover, to ensure that behavioral data from the Severe HI subset in Study 1 was comparable to data from the overall HI group in Study 1, we used the Severe sub-groupings to re-analyze average Trial 1 errors 1 at each Week, and compared findings to results with all HI subjects. These analyses confirmed that the subset of “severely” damaged HI animals showed the same pattern of errors across the task as the HI group overall.

### 3.2. Behavioral Results (Study 1 and 2)

#### 3.2.1. Study 1 and 2, Analysis of Control Trial Errors

For both Study 1 and Study 2, analysis of errors made during control trials (using Treatment as a between variable) confirmed a lack of differences between HI and shams. Both groups showed a consistent and equivalent number of errors across testing, and errors were much higher than for test trials. This confirmed that (1) animals were not using some other cue (visual, odor) to find the goal, and (2) HI animals were equally able to traverse the maze compared to shams.

#### 3.2.2. Study 1, Analysis of Overall Errors

A multi-variate analysis of mean weekly errors from Study 1 revealed no overall effects of Treatment, nor a Treatment × Condition interaction [(*F*(1,22) = 0.434, *p* > 0.05); (*F*(3,66) = 0.487, *p* > 0.05), respectively]. We did, however, find a significant effect of Condition (*F*(3,66) = 4.335, *p* < 0.05) and Week (*F*(1,22) = 23.354, *p* < 0.005). Therefore, based on a priori hypotheses, further planned comparisons were conducted as described below.

#### 3.2.3. Study 1, Analysis of Errors within Condition

**Figure 3:**
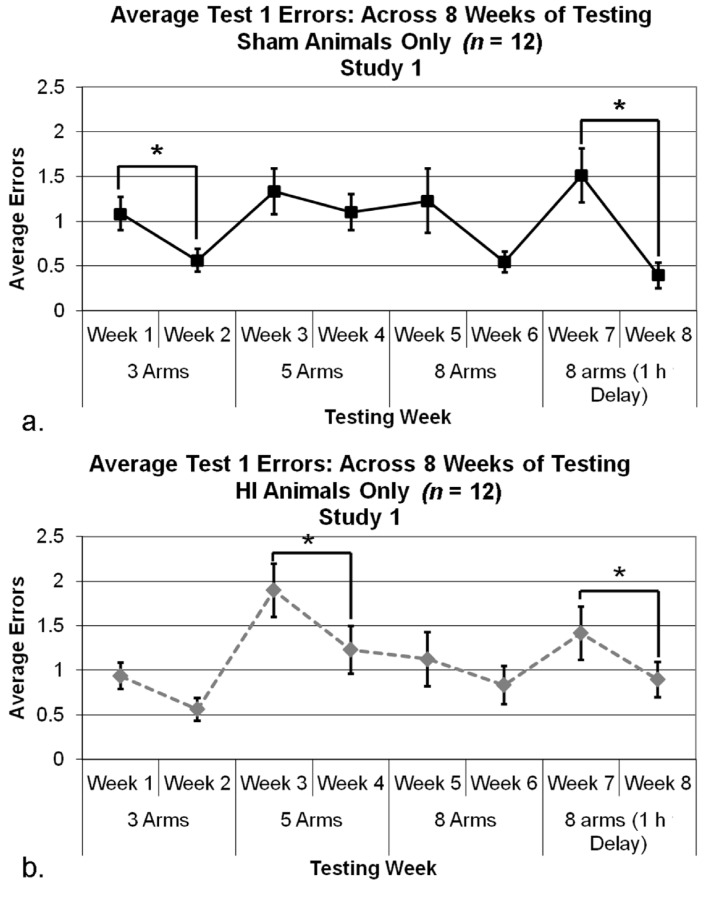
(**a**) A repeated measures ANOVA assessing Trial 1 errors in each Condition revealed a significant effect of Week in Condition 1 (* *p* < 0.05) and Condition 4 (* *p* < 0.05), indicating less errors made on the second Week of a Condition. (**b**) A similar analysis was done with HI animals and revealed a significant effect of Week in Condition 2 (* *p* < 0.05) and Condition 4 (* *p* < 0.05), indicated less errors (and evidence of learning) on the second week of a Condition.

Repeated measures ANOVAs were used to assess each Week (two levels) within each Condition, for each Treatment group (HI and sham) separately. For shams, a significant effect of Week was seen for Condition 1 (*F*(1,11) = 11.547, *p* < 0.05), and Condition 4 (*F*(1,11) = 7.237, *p* < 0.05) (see Figure 3a), indicating shams were able to learn (*i.e.*, fewer errors over time). Week comparisons for Condition 2 (*F*(1,11) = 0.805, *p* > 0.05) and Condition 3 (*F*(1,11) = 1.530, *p* > 0.05) did not show significant effects. For HI subjects, similar analyses revealed a significant effect of Week for Condition 2 (*F*(1,11) = 5.770, *p* < 0.05), and Condition 4 (*F*(1,11) = 5.074, *p* < 0.05; Figure 3b), indicating HI subjects were also learning the task and made significantly fewer errors over time. Further analyses of HI animals’ performance within remaining Conditions (1 and 3) revealed no significant effects of Week [(F(1,11) = 2.137, *p* > 0.05); (F(1,11) = 1.233, *p* > 0.050), respectively].

#### 3.2.4. Study 1, Analysis of Errors for Each Week, by Treatment

**Figure 4:**
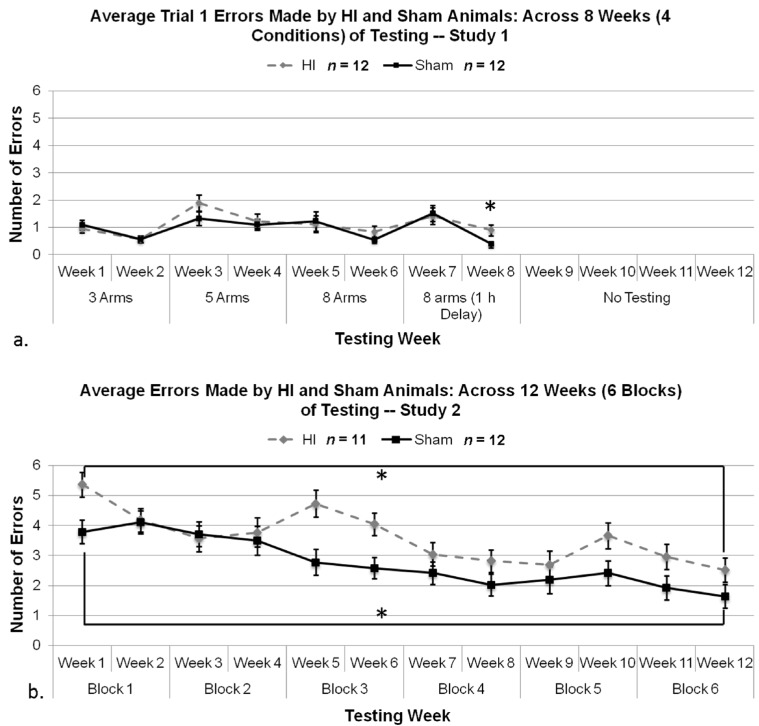
(**a**) A one tailed independent samples *t*-test using Tukey’s test to correct for multiple comparisons revealed a significant effect of Treatment (* *p* < 0.05) in Study 1, with HI animals making significantly more errors than sham animals on Week 8. (**b**) A repeated measures ANOVA revealed a significant overall Treatment effect on mean errors made (*p* < 0.05), and paired samples *t*-test between Block 1 and 6 for HI (* *p* < 0.05) and sham animals (* *p* < 0.01) revealed significant differences in regards to average errors made in a test trial (*i.e.*, learning).

Based on the main effect of Condition as described in Section 3.2.2, coupled with a priori hypotheses, an additional series of *t*-tests were performed using Tukey’s test to correct for multiple comparisons [108]. Results of a subsequent series of *t*-tests comparing mean errors for each Week of testing as a function of Treatment revealed no effect for Weeks 1 through 7, with HI and Shams performing comparably [(*q*(22) = −0.891, *p* > 0.05); (*q*(22) = 0.180, *p* > 0.05); (*q*(22) = −2.369, *p* > 0.05); (*q*(22) = −0.820, *p* > 0.05); (*q*(22) = −0.455, *p* > 0.05); (*q*(22) = −1.547, *p* > 0.05); (*q*(22) = −0.439, *p* > 0.05), respectively; see Figure 4a]. However, during the last Week of testing, with the implementation of a one-hour delay, an independent samples *t*-test using Tukey’s *post-hoc* test to correct for multiple comparisons did reveal a small but significant effect of Treatment, (*q*(22) = −2.50, *p* < 0.05, 1-tailed) with HI animals making significantly more errors than sham animals (Figure 4a).

#### 3.2.5. Study 1, Re-Analysis of Errors for Each Week, by Treatment (Severe and Mild HI Subsets)

**Figure 5:**
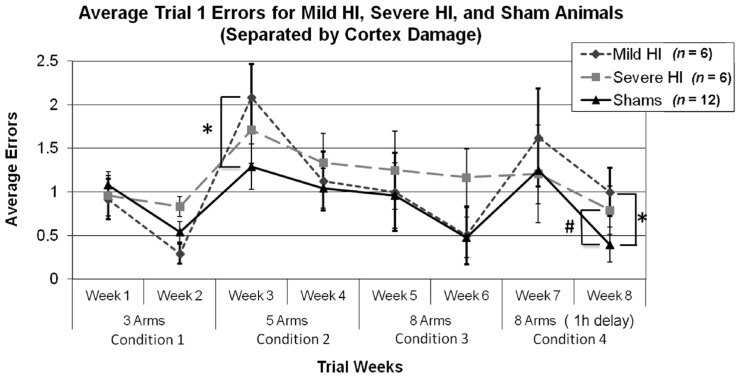
Re-analysis of errors by week for Study 1, using sub-groupings of Mild and Severe HI *versus* shams. Results were similar to overall analysis, with near-effects seen for both Mild and Severe sub-groups at Week 8 by way of one-way ANOVA, corrected for multiple comparisons using a Tukey’s *post-hoc* test (* *p* = 0.05, # *p* = 0.1, respectively). Interestingly, we did find an additional significant effect at Week 3, but this was seen for the Mild HI *versus* Sham (as evidenced by a Tukey’s *post hoc* analysis; * *p* < 0.05, one-tail), supporting the view that the lack of effects seen at earlier (easier) tasks in Study 1 was *not* a reflection of lesser damage in HI subjects as compared to Study 2.

Importantly, analyses of mean errors using all three Treatment groups revealed a similar pattern to that seen for the HI group as a whole (Figure 5). That is, an overall ANOVA (later corrected by using a Tukey’s test) for Treatment (three levels), Condition (four levels) and Week (two levels) revealed a significant effect of Condition (*F*(3,63) = 4.783, *p* < 0.05), Week (*F*(7,147) = 3.772, *p* < 0.005), and Condition × Week interaction (*F*(21,441) = 3.799, *p* < 0.005). A further one-way Week (8) × Treatment (3) ANOVA revealed a trend (near-significant) effect on Week 8 (*F(*2,21) = 1.652, *p* = 0.1, one-tailed), with a Tukey’s *post hoc* test revealing a significant effect between Mild HI and Sham (*p* = 0.05, one-tailed) and a near significant effect between Severe HI and Sham (*p* = 0.1, one tailed), with both HI groups showing a trend towards fewer errors. However, the small number of animals in each group (*n* = 6 in each group) likely contributed to the marginal effect in Severe HI animals on Week 8. Interestingly, the one-way ANOVA also showed a marginal effect on Week 3 (Condition 2, HI worse than sham; *F*(2,21) = 1.638, *p* = 0.1, one-tailed), but a Tukey’s *post hoc* test revealed that this was seen for the *Mild* HI *versus* Sham, and not Severe *versus* Sham comparison (*p* < 0.05, one-tailed). Overall, this re-analysis supports our results in showing that behavioral deficits on easier tasks in Study 1 do not emerge, even when examining a sub-set of animals selected for severe cortical atrophy, with anatomic damage comparable to that of Study 2 (Figure 2).

#### 3.2.6. Study 2, Analysis of Errors Overall

Results of a 6 (Block) × 2 (Treatment) repeated measure ANOVA revealed a significant Block main effect (*F*(5,105) = 17.045, *p* < 0.001), indicating changing performance over blocks of testing (*i.e.*, learning) in both groups. There was also a significant Block × Treatment interaction (*F*(5,105) = 1.881, *p* = 0.05, 1-tailed), which allowed us to further perform paired samples *t*-tests for HI and sham animals to assess average errors made in the first *versus* last block of testing. Analyses revealed significant effects for both shams (*t*(11) = 11.191, *p* < 0.001) and for HI (*t*(10) = 3.972, *p* < 0.005) (Figure 4b), indicating that both Treatment groups showed significant improvement (learning) on the task by the last block of testing.

Additionally, we found a significant Treatment main effect (*F*(1,21) = 8.028, *p* < 0.05), with HI animals making significantly more errors overall than sham, throughout (Figure 4b).

#### 3.2.7. Study 1 and 2, Analysis of Errors in Weeks 7 and 8 by Treatment and Study

**Figure 6:**
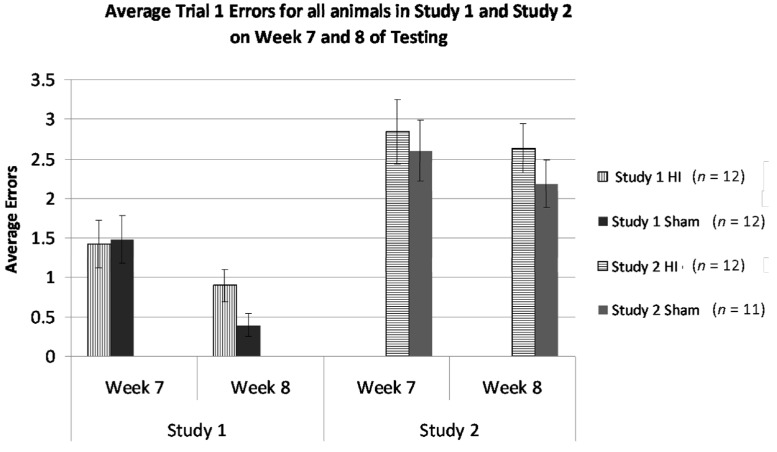
Bar-chart comparison of mean errors at Weeks 7 and 8 (using eight arms open, with delay), for HI *versus* sham, and across Study 1 and 2. Notably, these scores were attained after a comparable duration of testing (though using different lead-in tasks), and the tasks employed on Week 7 and 8 were largely identical across studies. Regardless of similar testing duration and similar parameters in these weeks, an overall ANOVA revealed a significant effect of Week (*p* < 0.005) and Study (*p* < 0.001). Note whereas HI subjects in Study 1 were making between 1 and 1.5 mean errors, HI subjects in Study 2 (who had not received progressive training) were making around three errors on the same task.

Due to specific cross-study comparability in task in Weeks 7 and 8, we made a direct statistical comparison (using multi-variate ANOVAs) of mean errors for sham and HI rats within this window, as a function of Study. This comparison was thought to reveal any effects of prior differential experiences, since the subjects received: (1) the same duration (six weeks) of prior testing (albeit it under different conditions); and (2) are now being tested under essentially the same conditions. An overall ANOVA using Study (between, two levels), Treatment (between, two levels), and Week (within, two levels) revealed a significant effect of Week (*F*(1,43) = 9.976, *p* < 0.005), as well as a significant effect of Study (*F*(1,43) = 25.686, *p* < 0.001, see Figure 6). This indicates that performance for HI and sham animals over Week 7 and 8 were different, regardless of Study and that performance by HI and sham animals was significantly different between Studies, where overall, animals in Study 1 were performing better than animals in Study 2. This can be attributed to the repetition of test trials in Study 1, as well as the gradual increase of task difficulty.

#### 3.2.8. Study 1, Analysis of Mean Latency per Choice, by Treatment

An overall ANOVA using Treatment (two levels; HI and sham), Condition (four levels) and Week (two levels) was used to assess average latency per choice (as above, only for Trial 1 errors). Initially, we found a Condition × Treatment interaction (*F*(3,66) = 4.279, *p* < 0.05), as well as main effects of Condition (*F*(3,66) = 15.131, *p* < 0.05) and Week (*F*(1,22) = 111.040, *p* < 0.05). We also found a Condition × Treatment × Week interaction (*F*(3,66) = 3.218, *p* < 0.05), thereby justifying the examination of Treatment effects on latency within each Week separately.

**Figure 7:**
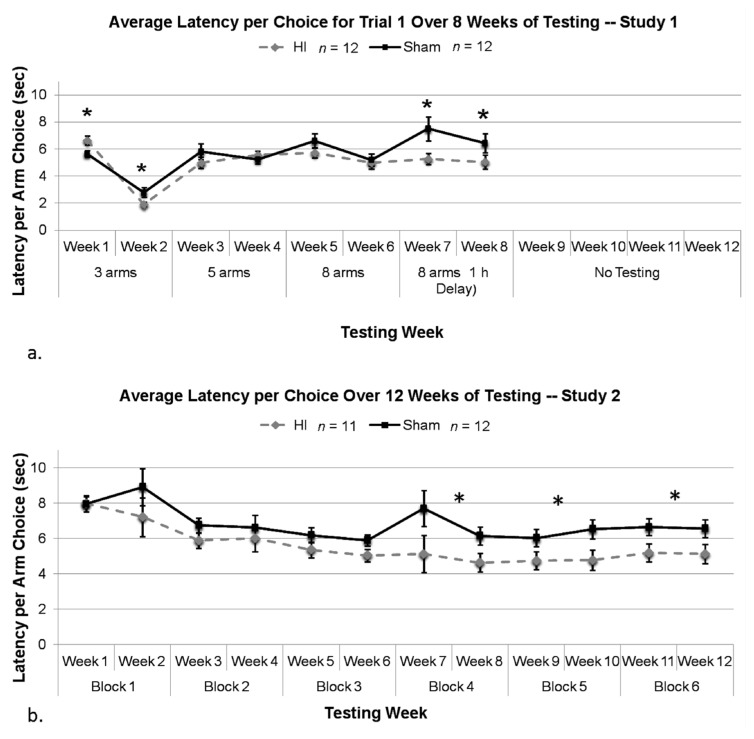
(**a**) We found a significant effect of Treatment, with HIs taking *more* time to make an arm choice on Trial 1 during the first week of testing only (* *p* < 0.05) only. On Weeks two and seven only, conversely, *t*-tests corrected for multiple comparisons by Tukey’s test revealed HIs were taking significantly *less* time to make an arm choice than shams (* *p* < 0.05), and analysis of Week 8 also revealed a significant effect for HIs to take less time than shams to make an arm choice (* *p* < 0.05). (**b**) A repeated measures ANOVA revealed a significant overall Treatment effect in regards to average latency to first arm choice (*p* < 0.05). Individual *t*-tests corrected for multiple comparisons via Tukey’s test for each block revealed a Treatment effect for Block 4 (* *p* < 0.05), Block 5 (* *p* < 0.05) and Block 6 (* *p* < 0.05).

Using a series of two-tailed *t*-tests which were corrected for multiple comparisons using Tukey’s test, we found a significant effect of Treatment in Week 1, with HI animals taking *more* time to make an arm choice as compared to shams [Condition 1, Week 1; (*q*(22) = −3.415, *p* < 0.05), Figure 7a]. Interestingly, on Weeks 2 (second week of Condition 1) and 7 (first week of Condition 4), a two tailed *t*-test combined with a Tukey’s test to correct for multiple comparisons revealed that HI animals were taking significantly less time to make an arm choice than shams [(*q*(22) = 3.259, *p* < 0.05), (*q*(22) = 3.246, *p* < 0.05), respectively; Figure 7a]. Analysis of Week 8 also revealed a significant effect for HI animals to make faster arm choices than shams (*q*(22) = 2.292, *p* < 0.05). All other Weeks (3–6) did not yield significant Treatment effects [(*q*(22) = −1.647, *p* > 0.05); (*q*(22) = −1.031, *p* > 0.05); (*q*(22) = 1.852, *p* > 0.05); (*q*(22) = 0.483, *p* > 0.05), respectively].

#### 3.2.9. Study 2, Analysis of Mean Latency per Choice, by Treatment

Results of a 6 (Block) × 2 (Treatment) repeated measures ANOVA looking at average latency per choice revealed a significant effect of Block (*F*(5,105) = 9.224, *p* < 0.001), as well as a significant overall Treatment effect (*F*(1,21) = 7.181, *p* < 0.05; Figure 7b). This indicates that both groups were making arm choices faster over weeks of testing, and that HI animals were taking significantly less time to make an arm choice compared to sham animals throughout. Further individual *t*-tests (further correct with Tukey’s test) revealed a significant Treatment effect on Block 4 (*q*(21) = 3.673, *p* < 0.05), Block 5 (*q*(21) = 3.240, *p* < 0.05), and Block 6 (*q*(21) = 3.613, *p* < 0.05), with HI animals making faster arm choices.

### 3.3. Discussion

Both term and preterm HI populations show heightened incidence for an array of cognitive and behavioral deficits, including language impairments [32,33,35,36,37,109,110], attentional problems associated with ADHD [42,43,44,45,46,47,54,63,111] and memory impairments [33,38,39,40,56,57,64,112,113]. In the current series of studies, we focus specifically on working memory impairments associated with neonatal HI injury in a rat model. This emphasis reflects the fact that memory impairments are an extremely common outcome in clinical HI populations, and may relate to poor academic achievement, as well as lower IQ scores in childhood [55,56,57,102,103,104,107]. HI-associated memory impairments may also co-occur with additional disabilities, for example in executive functioning and/or attention [51,52,57,64,65,67,105,106,112,113]. Overall, memory deficits appear to form a core impediment to educational success for neonatal HI populations.

The current set of studies specifically sought to detail the nature of HI-associated working memory deficits, using a rodent model of P7 HI injury, which simulates term HIE in humans. Our study design was guided by prior evidence of a P7 HI associated deficit on Morris water maze tasks in rodents [21,74,77,78,79,80,81,82,83,84,101], as well as deficits seen for HI rats on a standard radial arm land maze paradigm [86,87,89].

In brief, our results revealed the following. (1) Subjects with P7 HI damage can learn, regardless of task order and difficulty level. In fact, our evidence suggests that HI rats showed about the same degree of learning on a working memory task as shams (as indexed by decreasing errors over time; Figure 4); (2) Deficits in memory processes associated with HI were far more apparent on tasks with a high working memory demand (e.g., tasks that employed a delay interval). Indeed, despite evidence of learning, HI performance in Study 2 was still significantly worse than shams after 12 weeks of training; (3) HI subjects showed a reduction in latencies despite more errors made (*i.e.*, latencies were shortened under the same conditions where errors were high), likely reflecting impulsive tendencies when confronted with difficult tasks. Thus, evidence of impulsivity was much more pronounced on the more difficult task (Study 2; Figure 7b) as compared to the graduated one (Study 1), with HI response latencies remaining shorter than shams across 12 weeks of training in Study 2. Conversely, response latencies for HI and sham rats were often seen to merge on easier tasks in Study 1 (Figure 7a); (4) All other factors being equal, HI rats with prior experience on a graduated multi-trial task performed significantly better on a high-demand working memory task when compared to similar HI rats introduced directly to the more difficult paradigm (Figure 6)*.* The implications of these findings are discussed further below.

#### 3.3.1. HI Rats Still Show Learning, Even When the Task is Difficult

This finding is perhaps not surprising, since at-risk children are clearly able to learn, albeit at a slower or reduced-grade-level rate [114]. For example, Ritter and colleagues conducted a study specifically looking at whether preterm infants (at-risk for an HI injury) showed prominent executive functioning/memory impairments that persisted or if preterm infants were able to “catch up” with term controls with increasing age. We believe that these findings can be related to our “term” HI model since both preterm and term children at-risk for HI injury show similar behavioral impairments. Thus, results of the study were congruent with the animal results of the current study, in which preterm infants are more likely to show a delay in learning, rather than a deficit. In other words, rather than displaying a consistent learning deficit over time, children are able to learn a difficult task but at a slower rate. This finding is also in parallel with other clinical literature where learning was apparent in preterm children (though not as quickly as typically developing children) based on verbal IQ scores and attentional impairments [115,116]. Moreover, in a longitudinal study by Cserjesi and colleagues, children born prematurely display learning impairments in adolescence but these impairments can be attenuated, regardless of the increasing cognitive demands as children age [27].

Indeed, future research into remediation strategies should capitalize on these observations, especially since research has suggested that prematurely born children activate different brain areas than term children as task difficulty increases [117]. Since preterm children do show learning despite being at risk for an HI injury, it is important to ameliorate these subtle learning impairments in addition to mitigating more severe cognitive and behavioral impairments. Specifically, tailored interventions should be developed early on to minimize the adverse cognitive consequences of premature birth and to reduce the impact of early learning challenges on educational performance and learning motivation [118].

#### 3.3.2. HI Deficits are More Robust on High Memory Demand Tasks

The fact that memory deficits in HI rats are exacerbated on more difficult memory tasks is also perhaps unsurprising, and parallels at least one report showing that premature children (*i.e.*, at risk for HI insult) show memory impairments only when task difficulty increased—a finding interpreted to reflect an inability to initiate and sustain organizational and complex memory strategies when under high demand [68]. Furthermore, preterm children at risk for an HI injury show robust impairments on backwards phases of working memory tasks compared to typically developing term children, indicating prematurely born children display deficits on a difficult memory task [66]. These findings parallel supplementary research comparing fMRIs of preterm children *versus* term children, in which preterm adolescents recruit a wider array of neural networks than full term adolescents [117,119]. Specifically, neuropathological and behavioral data both indicate that when memory demands are high, the preterm infant’s neural mechanisms responsible for memory and executive functioning are overwhelmed and stressed, thereby compromising behavioral performance [68,120,121]. Similar findings also reveal that cognitive difficulties (*i.e.*, memory impairments) become more evident and stable later on in development as children reach school age. This is primarily due to the more complex memory dependent activities that occur once children reach adolescence and are required to sustain information for schooling [122]. Additionally, animal research has also supported this idea, pointing toward a similar explanation where early (easy) trials on a maze task yield similar performances from HI and sham animals, but as testing progresses and the task becomes more reliant on working memory (making the task more difficult), HI animals are unable to learn the task, whereas sham animals display learning [123].

#### 3.3.3. HI Rats Showed Reduced Latencies Compared to Shams Despite More Errors Made, and This Impulsivity was Strongly Related to Task Difficulty

The fact that we found evidence of impulsivity (*i.e.*, an ADHD characteristic) in both Study 1 and Study 2 is again consistent with human clinical data. That is, a common symptom in preterm children or children at risk for HI that are also diagnosed with ADHD is impulsivity/hyperactivity [32,42,43,48,49,111,124], and the prevalence for diagnosis is more common in males [125,126,127,128]. Our findings also support evidence from other animal studies looking at ADHD-like behaviors, for example in a CRT task. Here, studies have shown that on later trials with a delayed reward, hypoxia-exposed rats made significantly more lever presses than control rats [90]. Moreover, studies have shown that male rats with induced HI display greater impulsive symptoms as assessed by the 5CSRT task [129,130,131,132].

Interestingly, however, our results also revealed that indices of impulsivity were much greater when subjects were performing the more difficult task, and response latencies in HI rats continued to be significantly shorter than shams (despite worse performance) even after 12 weeks of training (Figure 7). Essentially, HI animals are more likely to “make errors quickly,” which appears consistent with the impulsiveness typically seen in children with ADHD (a common neurodevelopmental disorder seen in the clinical HI population). In contrast, sham animals take longer to make what is more frequently a correct choice. Interestingly, latencies for HI and sham rats were often seen to equalize on the easier versions of the task (Study 1; Figure 7), indicating that impulsivity in HI subjects may only be seen when the task is more difficult (*i.e.*, too difficult). These findings are relatively novel, and suggest that impulsivity scores could provide an indirect index of “degree of challenge” in educational efforts with HI populations. As an aside, it should be noted that impulsivity is not the only possible interpretation for short latencies, and latency data may be affected by other factors. However, given consistency with clinical data, the current interpretation is well supported.

#### 3.3.4. HI Rats Learn Much Better When Introduced to a High Memory-Demand Task through Gradual Training, as Opposed to Immediate Introduction

These results may appear intuitive, yet are relatively novel in the animal model literature, as well as in the clinical medical setting. That is, relatively little focus is placed on tailored, graduated, progressive task introduction when testing impaired models in experimental research (although this topic may well receive more attention in the educational setting). In fact, many of the novel computer-based educational intervention programs commercially available do employ “staircase procedures,” wherein difficulty of problems presented is driven explicitly by performance, providing an individually tailored experience. However, overall, it appears this issue has received relatively little notice as a mechanism that might explicitly and particularly benefit clinical populations with educational challenges (such as children with neonatal HI).

#### 3.3.5. Underlying Neuropathology for Memory Deficits and Impulsivity

Neuropathological indices in both studies indicated brain damage following HI, specifically in the form of reduced cortical and hippocampal volume, with significant damage seen in severely injured HI animals in Study 1, and in all HI animals in Study 2 (Figure 2). Other labs have also reported smaller volumes of the hippocampus and cortex in neonatal HI rats, attributing these effects in part to excitotoxicity in these gray matter areas following an HI insult [1,6,18,21,24,133]. Of particular importance to the current compilation of studies is damage to the hippocampus, as this structure is highly critical to executive functioning and memory [38,68,112]. Additionally, it has been shown that the cortex (particularly the dorsolateral prefrontal area) is linked to spatial working memory performance in humans [53,68]. Therefore, it is not surprising that severe HI animals in Study 1 and all HI animals in Study 2 (with significant damage in these areas) also showed deficits on spatially related learning/memory.

In terms of human clinical data, research has consistently pointed towards an essential role of the hippocampus and cortical structures in regulating normal cognitive functioning, in particular memory and attentional indices [39]. In regards to working memory, the prefrontal cortex has been shown to play a specific role [39,40,56,134,135]. Consequently, if these areas are damaged due to an HI insult (in the form of volume reduction), memory deficits can potentially emerge. Additionally, executive functioning and attentional impairments (*i.e.*, impulsivity) often co-occur with memory impairments, indicating the reliance on similar neural substrates [44,60,136]. Evidently, clinical and animal literature report coinciding brain areas related to memory and attention.

## 4. Conclusions

In conclusion, these studies reveal that HI animals *can* learn a difficult memory task (despite worse performance than shams), and moreover, can learn the task much more easily when it is introduced in a graduated and repetitive (multi-trial) fashion. Also, evidence of impulsivity is more robust on a high-demand task introduced without preface (*i.e.*, response latencies remained shorter than for shams across testing on the difficult task, but often came together on the easier tasks), and this impulsivity persists despite improved performance. Future research could investigate whether direct measures of impulsivity (as measured by latency to respond) could be used to index and adjust task difficulty when working with clinically affected populations, in order to tailor tasks more directly to the capabilities of children to learn and perform. That is, impulsivity measures may provide an indirect index that children are overly challenged, and that task parameters should be adjusted to a slightly lower level of demand. Overall, our findings suggest strongly that educational efforts with clinical HI populations (*i.e.*, children exhibiting learning impairments associated with early brain damage) could benefit tremendously from enhanced efforts to implement individualized task calibration, with progressive introduction of more difficult levels of work. Indeed, our results suggest that clinical populations could well reach performance levels comparable to typical populations if provided these task-based adjustments.
